# Neural Correlates of Advantageous and Disadvantageous Inequity in Sharing Decisions

**DOI:** 10.1371/journal.pone.0107996

**Published:** 2014-09-19

**Authors:** Berna Güroğlu, Geert-Jan Will, Eveline A. Crone

**Affiliations:** 1 Institute of Psychology, Leiden University, Leiden, the Netherlands; 2 Leiden Institute for Brain and Cognition (LIBC), Leiden University, Leiden, the Netherlands; University of Pennsylvania, United States of America

## Abstract

Humans have a strong preference for fair distributions of resources. Neuroimaging studies have shown that being treated unfairly coincides with activation in brain regions involved in signaling conflict and negative affect. Less is known about neural responses involved in violating a fairness norm ourselves. Here, we investigated the neural patterns associated with inequity, where participants were asked to choose between an equal split of money and an unequal split that could either maximize their own (advantageous inequity) or another person’s (disadvantageous inequity) earnings. Choosing to divide money unequally, irrespective who benefited from the unequal distribution, was associated with activity in the dorsal anterior cingulate cortex, anterior insula and the dorsolateral prefrontal cortex. Inequity choices that maximized another person’s profits were further associated with activity in the ventral striatum and ventromedial prefrontal cortex. Taken together, our findings show evidence of a common neural pattern associated with both advantageous and disadvantageous inequity in sharing decisions and additional recruitment of neural circuitry previously linked to the computation of subjective value and reward when violating a fairness norm at the benefit of someone else.

## Introduction

Although economic models assume that the maximization of personal gains is the main motivation when distributing resources, investigations of actual decision-making have shown that fairness concerns play an important role in social interactions [1]–[6]. Indeed, the evidence is overwhelming: people have a preference for fair outcomes and, all else being equal, acting fairly is generally the expected social norm [1]–[8] and equality is often used as a cognitive heuristic in decision-making [9]. In search of proximate mechanisms it has been shown that equal distributions are perceived as rewarding, both indicated by self-reported ratings of fair divisions of resources as well as reward-related neural activation patterns associated with these choices [7], [8], [10], [11]. Further, being treated unfairly leads to anger [10]–[14] and has been associated with activation of neural networks involved in conflict and negative affect [12]–[16]. Finally, when confronted with unfair treatment and given the power to retaliate, people generally reject inequitable distributions of resources, even when this is costly for them [2], [15], [16].

Despite this strong preference for equity and the aversion towards inequity, people often make inequity choices, such as when inequity is more advantageous for the self. For example, people aim to increase relative advantage over others [2], [17], [18] and when a high social position is experimentally induced they become more selfish and display higher levels of immoral behavior, such as cheating and lying [19]–[22]. It is thus crucial to gain a better understanding of the neural mechanisms underlying inequity decisions in order to better understand when and why we decide to divide resources in an unequal fashion. The current study aimed to investigate the neural responses associated with inequity in sharing decisions when maximization of outcomes for the self or another person is in conflict with the equity norm.

Using allocation tasks such as the “Dictator Game” where participants divide a certain amount of rewards (i.e., the stake) between themselves and another player without sanctions or reputation-related consequences, many studies have shown that people often give away a nontrivial amount of the stake to anonymous others, with an equitable 50–50 split being the most frequent allocation [17], [18], [21], [23], [24]. Nonetheless, such a preference for fairness is highly sensitive to different aspects of the (social) context in which they occur [19], [21], [22], [25], [26]. For example, a preference for equity decreases when the costs of establishing equal outcomes increase, supporting the crucial role of self-outcome maximization in fairness considerations. Furthermore, people seem to be less tolerant to receiving less than other people (i.e., disadvantageous inequity) compared to receiving more than others (i.e., advantageous inequity) [7], [12]–[14], [21], [23], [24], [27]. In other words, fairness considerations are not solely shaped by other-regarding preferences and prosocial intentions, but also by self-outcome maximization and aversion to disadvantageous inequity [12], [25], [26].

Studies investigating the neural mechanisms associated with inequity have predominantly focused on the *perception and receipt* of unfair treatment [7], [12]–[14], [27], [28]. These studies have consistently shown involvement of the dorsal anterior cingulate cortex (dACC) and the anterior insula in perceiving unfairness. Interestingly, studies have shown heightened anterior insula activity when people themselves are the target of unfair treatment [12] and when they see someone else receiving an unfair offer [28]. Based on anterior insula’s domain general role in providing anticipatory emotional signals in decision-making [29]–[31] and the ACC and insula’s involvement in neural representations of bodily arousal states [29], [32]–[34], it has been argued that the ACC and anterior insula play an important role in guiding our social behavior to follow social norms [35]. Behaviors in response to unfairness have been consistently associated with activation in the dorsolateral prefrontal cortex (dlPFC), which has been suggested to reflect increased regulation of a default prepotent reaction to unfair offers [13], [27], [36]–[40]. Although these findings overall support the idea that equity is perceived as a social norm, fewer studies have investigated how neural responses to unfairness might be different when *making* inequity decisions. Two studies investigating allocation of resources to others who had previously excluded the participants from a social interaction have shown the involvement of the ACC – insula network when sharing unequally with those excluders [41], [42]. In the current study, we aimed to investigate whether inequity choices are processed differently than equity choices and how this depends on the benefit for the self and the other. For this purpose, we investigated inequity choices in different experimental conditions that aimed to disentangle inequity that is advantageous for the self from inequity that is advantageous for another person (while leaving the decision-maker’s own outcome unaffected).

First, based on previous findings, we expected higher insula and dACC activity when making inequity choices in general [29], [33]. A central question was whether the insula and dACC response subserves a general role through acting as a “social alarm system” that is activated in response to both advantageous and disadvantageous inequity, i.e. regardless of whether the participants themselves or another person benefits from the inequity. If equity were perceived as the social norm, we would expect higher levels of insula and dACC activity in making inequity choices across different conditions that differ in relative outcomes for self and other. However, if other-regarding (prosocial) outcomes were perceived as the social norm, we would expect increased levels of activation in this network when making choices that ensure equity, but also lead to less optimal outcomes for others.

Second, we tested the hypothesis that inequity choices that lead to benefit of others is associated with activation in neural circuitry previously linked to reward-processing. This hypothesis is based on prior studies wherein participants were the allocators of resources and that showed that neural regions implicated in the computation of subjective value and reward play an important role in resource distribution [29], [33]. Although the paradigms used in these studies differed considerably, these prior studies showed that reward-related brains regions [e.g. the striatum and ventromedial PFC (vmPFC)] were associated with choosing outcomes that maximized the amount of joint resources. However, paradigms in these studies did not investigate two core processes of fairness considerations, namely, choices that incur costs to the self [29] and a fair alternative to making inequity choices [33]. In the current study, we included similar experimental conditions that involved a fair alternative to inequity and that also differed in respective possible costs and benefits for the self and the other. We expected that choices indicating other-regarding preferences through a maximization of the other’s outcomes would result in increased activation in reward-related brain regions, such as the striatum and the vmPFC.

## Methods

### Participants and procedure

Twenty-eight young adults (*M* = 20.7 years, *SD* = 1.91; 11 male) were recruited through local advertisements. All participants were right-handed and did not report any contraindications for fMRI. Before scanning participants were familiarized with the scanner environment using a mock scanner. After scanning, they filled out a battery of questionnaires, and received €25 for their participation and an additional amount of money, which was told to be determined by their decisions in the allocation games. In reality everyone received an additional €2. The current study was conducted in accordance with the ethical standards of the American Psychological Association as expressed in the Declaration of Helsinki. All participants provided written informed consent for the study. The study was approved by the Leiden University Medical Center (LUMC) ethics committee. A radiologist reviewed all anatomical scans; no anomalies were reported.

### fMRI task description

Participants played the role of the allocator in a set of three modified dictator games [21]. In each game the participants were asked to distribute coins between themselves and an anonymous other player based on preset dichotomous choices. One of the two options was always a fair (equal) distribution of coins, i.e. one coin for the self and one coin for the other (1/1). The alternative distribution in the three games were as follows: i) one coin for the self and zero coins for the other (i.e., 1/0) in the *Advantageous Competitive Inequity* game, where the inequity choice maximized the difference between self and other without gains relative to the equity choice, ii) two coins for the self and zero coins for the other (i.e., 2/0) in the *Advantageous Self-maximizing Inequity* game, where the inequity choice maximized outcomes for the self, and iii) one coin for the self and two coins for the other (i.e., 1/2) in the *Disadvantageous Prosocial Inequity* game, where the inequity choice signified other-regarding (i.e., prosocial) concerns.

**Figure 1:**
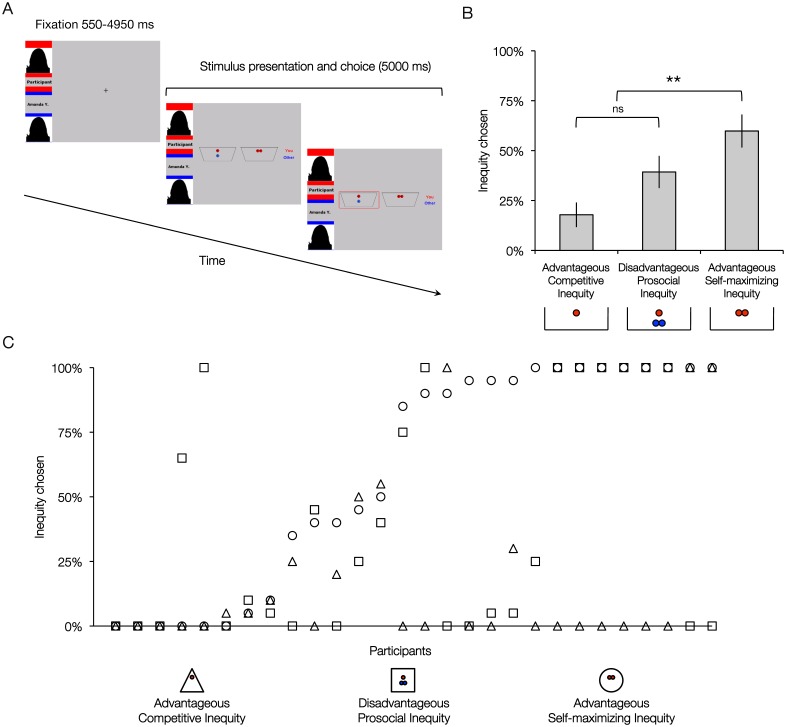
Visual display of the fMRI task and frequency of inequity choices. (A) Visual display of events presented in the one trial of the fMRI task. Each trial started with a jittered fixation cross lasting 550–4950 ms. The following screen displayed the name of the participant in red (here ‘Participant’) and the name of the recipient (here ‘Amanda Y.’). This screen also presented the available choice options for distributing the coins (here Advantageous Self-Maximizing Inequity game; 1/1 vs 2/0) with red and blue coins indicating the share for the participant and the recipient, respectively. The participant had a maximum response time of 4000 ms to make a choice. Upon response, the chosen distribution was encircled in red (here 1/1) until the end of the 5000 ms. (B) Percentage of inequity choices made in each of the three games. **p<.001, *p<.05. (C) Percentage of inequity choices made by each participant in each of the three games.

Each trial started with a jittered fixation cross (mean = 1540 ms, min = 550 ms, max = 4950 ms; optimized with Opt-Seq2, surfer.nmr.mgh.harvard.edu/optseq/; [43]). On the left hand side of this screen, participants were also presented with the name of the other player (see Fig. 1A). This was followed by the decision screen where participants were presented with two distributions (i.e., two buckets with coins in them) they could choose between. In each distribution coins for the self were indicated in red and coins for the other were indicated in blue. Participants had 4000 ms to make a choice. Upon making a choice, the bucket of their choice was encircled in red and this was displayed until the end of 5000 ms in total. In case of no response within the 4000 ms period, participants were presented a screen with ‘Too late!’ for the duration of 1000 ms. Trials without a response consisted of less than 1% of all trials and were excluded from further analyses. Prior to scanning participants were provided with instructions (see Text S1) and practiced the game (6 trials) on a computer. During the scanning session participants played a total of 60 trials, with 20 trials of each game, in randomized order. The location of the equal distribution was counterbalanced across trials. All trials were presented in one block lasting about 8 minutes.

On each trial, the first name and the first letter of the surname of both the participant and the recipient were displayed on screen to ensure anonymity, but also to emphasize the notion that participants would play each trial with a new player (see Figure 1A). Participants were told that random trials would be selected and their choices on these trials would determine their final earnings in the task. Prior to the experiment, participants were explained that the recipients were participants in the study and it was also emphasized that their decisions would have consequences for the other players’ earnings. None of the participants reported disbelief in the cover story that their offers influenced other players’ outcomes.

### fMRI data acquisition

Scanning was carried out at the University Medical Centre using a 3.0 T Philips Achieva. The scanning procedure included: i) a localizer scan, ii) T2*-weighted whole-brain echo planar images (EPI) measuring the bold-oxygen-level-dependent (BOLD) signal (TR = 2.2 s, TE = 30 ms, slice matrix = 80×80, slice thickness = 2.75 ms, slice gap = 0.28 mm, field of view (FOV) = 220 mm), iii) high-resolution T1- and T2- weighted matched bandwidth anatomical images with the same slice prescriptions as the EPIs. Functional data were acquired in a single functional run of 210 volumes; the first two volumes were discarded to allow for equilibration of T1 saturation effects. The task was programmed in E-prime and was projected onto a screen that was viewed through a mirror fastened upon the head coil assembly. Head movement was restricted by the use of foam inserts around the head.

### MRI data analysis

Image pre-processing and analysis was conducted using SPM8 software (www.fil.ion.ucl.ac.uk/spm). Pre-processing included slice-time correction, realignment, spatial normalization to EPI templates, and smoothing with a Gaussian filter of 8 mm full-width at half maximum. Movement parameters in all directions were below 1.08 mm for all participants and all scans. The fMRI time series were modeled by a series of events convolved with a canonical hemodynamic response function (HRF). The data were modeled at stimulus onset of the decision screen with zero duration and based on the game (3 levels: Advantageous Competitive Inequity, Advantageous Self-maximizing Inequity and Disadvantageous Prosocial Inequity) and participant’s choice (2 levels: equity or inequity), resulting in a 3×2 full factorial model that included six regressors. The participant-specific contrast images were obtained at the subject level and were then submitted to group level analyses at the second level, where participants served as a random effect in a repeated measures ANOVA. The full factorial ANOVA had an unbalanced design with varying number of participants in each cell of the model due to the fact that not all participants chose all options. The number of participants included in each cell of the design is as follows: Advantageous Competitive Inequity Game Equity choice (n = 25), Advantageous Competitive Inequity Game Inequity choice (n = 11), Advantageous Self-maximizing Inequity Game Equity choice (n = 19), Advantageous Self-maximizing Inequity Game Inequity choice (n = 22), Disadvantageous Prosocial Inequity Game Equity choice (n = 20), and Disadvantageous Prosocial Inequity Game Inequity choice (n = 18). We also conducted follow-up analyses examining the t-contrasts of Inequity > Equity for each game separately. Mean percentage of inequity offers in each game was used in regression analyses to test for brain-behavior relations in a GLM model based on the game (collapsed across choices; 3 levels: Advantageous Competitive Inequity, Advantageous Self-maximizing Inequity and Disadvantageous Prosocial Inequity). The fMRI analyses were conducted at the threshold of *p*<.001 uncorrected with a voxel threshold of 10 functional voxels to balance between Type 1 and Type 2 errors [44]. Regions of interest (ROI) analyses were further conducted on the regions obtained from the whole-brain analyses using the MARSBAR tool in SPM8 [45]. All results are reported in the MNI305 (Montreal Neurological Institute) stereotactic space.

## Results

### Behavioral results

An examination of response patterns of the participants showed that they had strong preferences for equity or inequity choices, which depended on the costs for self and other (see Table 1). A detailed overview of these choices per participant can be seen in Figure 1C. Percentage of inequity choices across the three conditions was compared using a repeated measure ANOVA, which yielded a significant main effect of Game (*F*(2,54) = 8.4, *p* = .001, η_p_^2^ = .24; Fig. 1B). Participants chose the inequity distribution more often in the Advantageous Self-maximizing Inequity condition (*M* = .60, *SD* = .43) than in the Disadvantageous Prosocial Inequity condition (*M* = .39, *SD* = .44; *F* (1, 27) = 4.90, *p*<.05, η_p_^2^ = .15) and in the Advantageous Competitive Inequity condition (*M* = .18, *SD* = .33; *F* (1, 27) = 21.98, *p*<.001, η_p_^2^ = .45). Inequity choices in the latter two conditions did not differ significantly from each other (*p* = .09, η_p_^2^ = .10). There was also a significant correlation between inequity choices in the Disadvantageous Prosocial Inequity and the Advantageous Competitive Inequity conditions (*r* (28) = −.41, *p*<.05).

**Table 1:** Frequency (and percentage) of participants making 100% equity, 100% inequity or both choices across the trials per game.

Game	100% Equity	100% Inequity	Both
*Disadvantageous Prosocial Inequity (1/2)*	10 (37.5%)	8 (28.6%)	10 (37.5%)
*Advantageous Competitive Inequity (1/0)*	17 (60.7%)	3 (10.7%)	8 (28.6%)
*Advantageous Self-maximizing Inequity (2/0)*	6 (21.4%)	9 (32.1%)	13 (46.4%)

### Neuroimaging results

**Figure 2:**
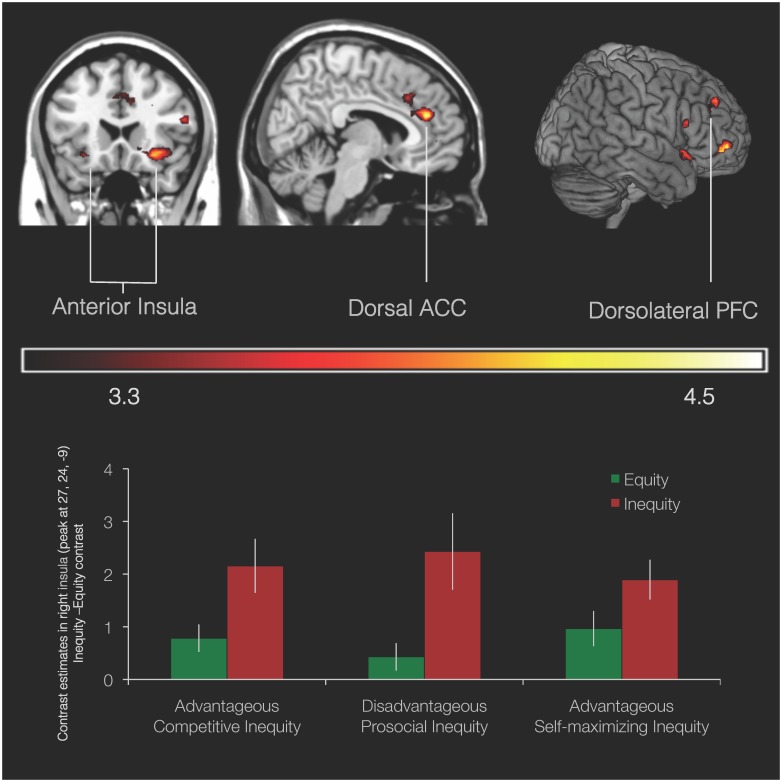
Neural network associated with inequity. Network of brain regions from the Inequity > Equity contrast in the 3 (Game) ×2 (Choice) full factorial ANOVA; *p*<.001, 10 voxel threshold. Bar graph displays contrast estimates obtained from ROI analysis in right anterior insula (MNI 27, 24, −9) for inequity and equity choices in the three conditions. Error bars indicate SEM.

In order to examine the neural correlates of equity and inequity choices, we conducted the Inequity > Equity and reverse contrasts within the 3 (Game) ×2 (Choice) ANOVA. The Inequity > Equity t-contrast revealed a network of regions comprising bilateral insula (x/y/z coordinates: −30, 21, −12; 19 voxels and 27, 24, −9; 95 voxels), right IFG (54, 21, 18; 12 voxels), dorsal ACC (6, 39, 21; 46 voxels and 0, 24, 36; 61 voxels), and dorsolateral (27, 45, 36; 22 voxels) and ventrolateral PFC (30, 54, −3; 49 voxels) (*t*(109) = 3.17; Figure 2; activation levels obtained from ROI analyses in right insula is plotted for demonstration purposes in a bar graph of activation per game and offer). The reverse contrast (Equity>Inequity) did not yield any clusters of activation and the game by choice interaction also did not result in significant activation. Thus, insula, ACC and dlPFC were activated in response to choosing an unequal distribution of resources, regardless of the consequences of this distribution for self or other in terms of maximizing outcomes or costs.

Next, in order to examine inequity related neural responses in more depth, we focused on the Inequity > Equity and reverse contrasts in the context of each of the three games separately using t-tests. The Equity > Inequity contrast did not yield activation in any of the three games. We also did not detect any regions for the Inequity > Equity contrasts in the Advantageous Competitive (n = 8) and the Advantageous Self-maximizing (n = 13) games at the chosen threshold, but note that the effects reported above are partially replicated at a more lenient threshold (see Table S1).

**Figure 3:**
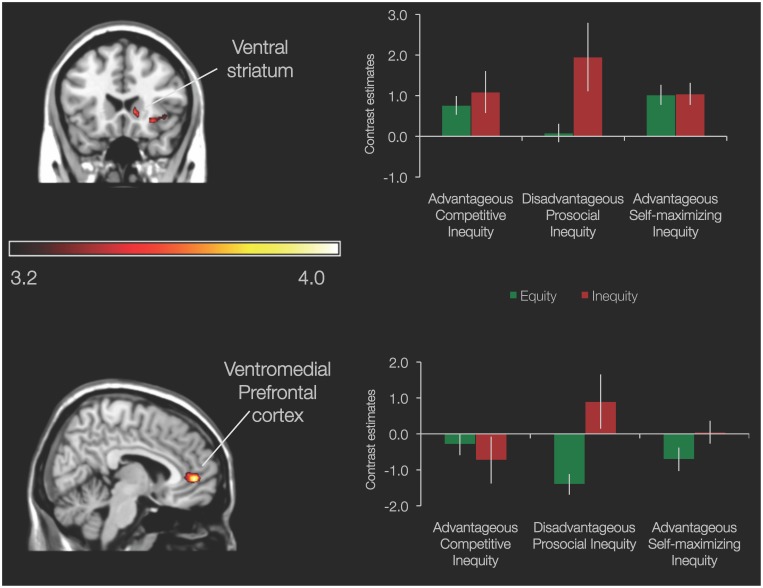
Neural network involved in Disadvantageous Prosocial Inequity. Ventral striatum (MNI 12, 21, 0) and ventromedial PFC (MNI 6, 48, 0) from the Inequity > Equity contrast in the Disadvantageous Prosocial Inequity condition; *p*<.001, 10 voxel threshold. Bar graphs display contrast estimates obtained from ROI analyses for inequity and equity choices in the three conditions. Error bars indicate SEM.

The Inequity > Equity contrast in the Disadvantageous Prosocial Inequity condition (n = 10) yielded increased activation in the vmPFC (6, 48, 0; 62 voxels), ventral striatum (12, 21, 0; 11 voxels), and right anterior insula (45, 15, −6; 53 voxels) during inequity choices than equity choices (Figure 3; activation levels obtained from ROI analyses in ventral striatum and vmPFC are plotted for demonstration purposes in a bar graph of activation per game and offer). Importantly, here the inequity choices were not only disadvantageous for the self relative to the other player, but also beneficial for the other player. Post-hoc ROI analyses showed that higher activation in these regions during inequity than equity was specific for the Disadvantageous Prosocial Inequity game; inequity and equity related activity in the Advantageous Competitive and Advantageous Self-maximizing Inequity games did not differ significantly in any of the regions (all *p*>.25).

**Figure 4:**
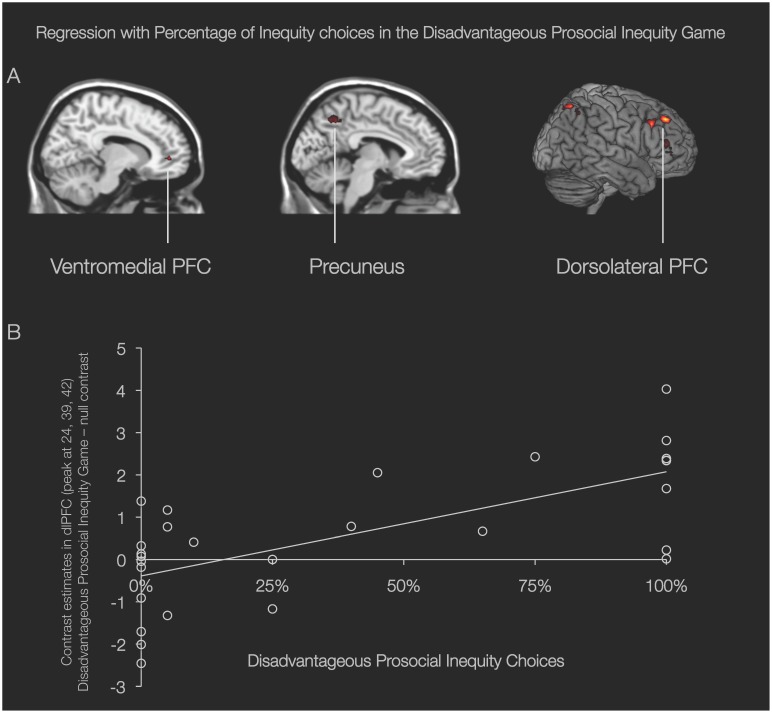
Neural network related to frequency of inequity choices. Brain regions from the regression of neural activity during the Disadvantageous Prosocial Inequity game with frequency of inequity choices. (A) Activation in the ventromedial PFC (MNI 15, 45, 0), precuneus (MNI -9, -57, -48), and dorsolateral prefrontal cortex (dlPFC; MNI 24, 39, 42) correlates positively with the frequency of inequity choices in the Disadvantageous Prosocial Inequity condition; *p*<.001, 10 voxel threshold. (B) Scatter plot displays contrast estimates for the Disadvantageous Prosocial Inequity condition on the y-axis and behavior (% inequity) on the x-axis (N = 28).

Finally, we examined brain-behavior relations by conducting whole-brain regressions where inequity choice frequency was included as a regressor in activations involved in the Disadvantageous Prosocial Inequity Game (collapsed across choices) – null contrast (n = 28). This approach enabled us to examine the relation between frequency of inequity choices and brain activation across the complete sample of 28 participants, whereas the previously reported inequity vs. equity and reverse contrasts could be examined only among the 10 participants who had made both equity and inequity choices in the Disadvantageous Prosocial Inequity condition. This analysis resulted in a set of regions in which activation correlated positively with inequity choices, including the precuneus (−9, −57, −48; 25 voxels), ventromedial PFC (15, 45, 0; 23 voxels), and dlPFC (MNI 24, 39, 42; 33 voxels) (Figure 4; the relation between DLPFC activation and frequency of inequity offers is demonstrated in a scatterplot). There was no activation in brain regions of interest in the brain-behavior correlations for the other two games (see Table S2).

## Discussion

The current study set out to investigate the common and distinct neural responses associated with inequity decisions involved in maximizing outcomes for the self or another person. Our behavioral results demonstrate that participants more often chose unequal distributions in situations where their own profits could be maximized relative to alternatives where they could maximize the other person’s profits. The neuroimaging findings showed that choosing inequity regardless of whether it entails benefits for the other is associated with increased activation in the anterior insula, dACC and dlPFC. In addition, decisions to distribute resources unequally, but in a way that benefits another person’s profits additionally coincided with increased activation in ventral striatum, vmPFC, precuneus and dlPFC. Taken together, our findings show that there is a common neural response to making advantageous and disadvantageous inequity choices, which resembles the pattern of neural activity previously associated with being treated unfairly [12]–[14], [28]. Furthermore, we show a distinct neural response associated with prosocial inequity, which suggests that violating a fairness norm in order to increase another person’s outcomes is processed differently on a neural level compared to selfish violations of a fairness norm.

Our behavioral findings show that participants adjusted their behavior depending on the available alternatives to an equal split. In doing so, it seems that different principles interact to guide decision-making when distributing resources: a social norm of equity, (possible) costs for the self, and a concern for outcomes of others *relative* to the self. Whereas an equal distribution was the most preferred option when it did not involve possible costs to the allocator (i.e., the participant), equal distributions became less preferred when it was costly to establish them. This finding is in line with previous studies on fairness preferences, which show that, although an equal split is used as a cognitive heuristic, contextual factors related to the relevance of self-interest systematically shifts preferences away from an equal split [9]. Preference for an equal distribution was not only influenced by *absolute* costs, as in the Advantageous Self-maximizing Inequity condition, but also in terms of *relative* costs compared to the other player, as in the Disadvantageous Prosocial Inequity condition. This latter finding demonstrates that a preference for equal outcomes does not necessarily have to be grounded in a prosocial motivation, but might also result from the desire to avoid receiving lower payoffs than another person [2], [28], [46], [47].

Neuroimaging results further show that there is a common neural response in dACC, bilateral anterior insula and dlPFC to both advantageous and disadvantageous inequity. This suggests that a general neural mechanism is implicated in signaling deviations from a fairness norm in sharing decisions, regardless of who benefits from the unequal distribution of goods. Our findings corroborate previous findings showing that both advantageous and disadvantageous inequity were associated with anterior insula activity [48] and a heightened medial frontal negativity [49], which has been interpreted as suggesting the involvement of the insula-ACC network in norm and associated expectancy violations. The dACC and the anterior insula are part of a “salience network” that serves an important domain general role in integrating cognitive and emotional signals when processing motivationally salient information [47], [50]. Activation in this network has been associated with error processing [51], uncertainty [52], conflict [53] and violations of a social norms [28], [46], [47], [54], [55]. We extend previous research by showing that the insula and dACC are also activated when creating inequity in choices that involve possible costs to the self and a fair alternative to inequity, both of which are core components of fairness considerations previously not investigated using fMRI.

Increased dlPFC activity during both advantageous and disadvantageous inquity choices relative to equity choices fits with findings from a recent study showing dlPFC involvement in both advantageous and disadvantageous inequity in a game in which participants received less or more money than another person after performing a perceptual task [56]. Based on its role in cognitive control and goal-directed behavior it has been argued that dlPFC activity in social decision-making tasks reflects increased control over prepotent responses that are aimed to maximize self-gain [27], [36], [39], [57], [58]. Our results suggest that dlPFC activity might reflect higher levels of executive control required to violate a salient social norm regardless of whether this maximizes gains for the self or someone else. The notion that this is not restricted to maximizing outcomes for the self was supported by our individual differences analyses that showed that participants who more often chose outcomes that maximize the profits of the other over an equal distribution recruit the dlPFC to a greater extent when doing so.

In addition to a common neural pattern associated with inequity, we also found that violations of a fairness norm in the Disadvantageous Prosocial Inequity condition were associated with activation in the striatum and the vmPFC. Activation in these regions associated with such prosocial behavior that leads to better outcomes for another person is in line with prior findings showing that the striatum not only responds to primary rewards, but also to social rewards such as charitable donations [59], [60], maximizing another person’s outcomes [33], [61], [62], and mutual cooperation in a prisoner’s dilemma paradigm [21], [26], [61], [63]. Moreover, individual differences analyses showed that the more frequent people showed this other-outcome maximizing behavior, the more they activated the vmPFC and the precuneus. The vmPFC is not only important for the encoding the subjective value of rewards [64], [65], but is also part of a network, including the precuneus, dorsomedial prefrontal cortex and the temporo-parietal junction [66], [67] important for mental state-reasoning [14], [41], [68], [69] and perspective-taking [60]. Moreover activation in the mPFC has been shown to be associated with processing one’s own and other people’s actions and intentions in economic games [61], [62]. Acting in a way that does not necessarily benefit outcomes for the self, but is beneficial to another person’s gains might thus possibly require increased levels of perspective-taking. It would be recommended for future studies to assess self-reported subjective value associated with individuals’ choices of advantageous and disadvantageous inequity in order to be able to examine how experience of reward is related to the neural signal associated with these choices.

Several limitations of the current study should be noted. One of the main challenges of the current research design is related to individual differences in observed behavior. As indicated by the behavioral patterns (see Figure 1C), the majority of participants were consistent in their choices within a certain condition, which might be considered desired given that this consistency reflects stable individual preferences and implies that participants did not choose randomly. However, this resulted in relatively small numbers of observations in several neuroimaging analyses where choice-related neural activation was examined based on contrasts of inequity versus equity choices per condition. For example, although there was a main effect of the Inequity > Equity contrast across conditions, these effects could not be observed when this contrast was examined per condition separately at the chosen threshold, but was only evident at more lenient threshold levels. In addition, the results may represent the neural activity of individuals who are ambiguous about equity choices, and in future research it should be examined whether these also represent choices of individuals with strict equity norms. Previous behavioral studies using the three allocation games have also examined profiles of individual behavior patterns [21], [26]. In the current study, our sample size did not allow us to examine the neural correlates of individual behavioral profiles. Future studies employing larger sample sizes should aim to examine individual differences in neural activation related to profiles of behavior.

The individual differences in behavior also resulted in an unbalanced design in our fMRI analysis. In other words, due to the fact that not all participants made all choices in each game, it was not possible to conduct a balanced full-factorial analysis with the same number of observations in each cell of the design. Future studies can aim to manipulate the study design in order to obtain a more balanced response pattern or, as indicated above, aim for larger sample sizes that will enable to examine individual differences based on choice profiles.

Furthermore, the current study did not employ self-report explicit measures about cognitive and affective processes related to making (inequity) choices. Future research should include measures about beliefs on fairness norms, affect related to inequity choices or autonomic measurements, such as heart rate, which can provide the researchers with additional measures in interpreting behavioral and neural findings.

The current results offer a number of avenues for future research. For example, our current design did not allow for a dissociation between joint-outcome maximization and maximization of another person’s outcomes in the disadvantageous inequity (1/2) choices. Future studies could include a condition where the 1/1 option is pitted against a 2/1 distribution, in which the latter choice would both be self- and joint outcome maximization [70]. A contrast between the 2/1 and 1/2 choices could disentangle joint outcome maximization from person-specific (self vs. other) outcome maximization. Furthermore, using the same set of three allocation tasks [21], [26] and other paradigms [71], [72] it has been shown that across development children and adolescents increasingly start enforcing equality between the ages of 3 and 13. Recent developmental work has shown that developmental changes in late maturing brain regions such as regions of the mentalizing network and the lateral PFC are associated with developmental increases in intentionality understanding and strategic considerations in fairness decisions [14], [37]. It would be of great interest to relate behavioral changes in both advantageous and disadvantageous inequity choices to brain development, because taking a developmental perspective has the potential to enhance not only our understanding of social development, but could also provide insights into adult social decision-making and its underlying mechanisms.

Taken together, the current results further inform our understanding of an important aspect of human social behavior, that is, when and why we decide to divide resources unequally. We show that violations of an equity norm, both with selfish (i.e., advantageous) and prosocial (i.e., disadvantageous) outcomes, are associated with a common neural response in the “salience network”. Furthermore, prosocial violations of a simple fairness norm were associated with activation in brain regions that code for primary and more complex social rewards [29], [33] and switching attention to another person’s perspective [60]–[62]. These findings show that neural networks implicated in social cognition, domain general cognitive functions and emotional processes are important for both following social norms and for violating such norms when these violations serve a more prosocial purpose than the norm itself.

## Supporting Information

Table S1 Regions of neural activation from the Inequity > Equity contrast per allocation game.
(PDF)

Table S2 Regions of neural activation from whole-brain regression analyses with frequency of inequity choices per game as a regressor.
(PDF)

Text S1 Task instructions for the allocation game.
(PDF)
