# Supplementary material for: Neural Correlates of Advantageous and Disadvantageous Inequity in Sharing Decisions
Source: PLoS One. 2014 Sep 19;9(9):e107996. doi: 10.1371/journal.pone.0107996 (PMC4169616; doi:10.1371/journal.pone.0107996)
Supplement: Table S1 — Regions of neural activation from the Inequity > Equity contrast per allocation game. (PDF) [file pone.0107996.s001.pdf]

Table S1. Regions of neural activation from the Inequity > Equity contrast per allocation game at the threshold of *p* < .005 uncorrected, > 10 voxels.

Anatomical region	L/R	Voxels	z	MNI coordinates		
				x	y	z
**Disadvantageous Prosocial Inequity condition: Inequity > Equity**						
Dorsolateral prefrontal cortex	L	24	3.49	-30	36	36
	R	28	3.47	27	42	36
Lateral prefrontal cortex	R	10	2.94	45	39	18
Ventromedial prefrontal cortex	-	238	3.99	6	48	0
Insula	R	209	3.69	45	15	-6
Ventral striatum			3.63	12	21	0
	L	111	3.37	-18	-3	-6
Precuneus	L	33	3.53	6	-42	45
	-	52	3.10	-6	-72	33
Inferior frontal gyrus	L	15	3.46	-54	15	18
	R	35	3.24	33	21	30
Temporoparietal junction	R	36	3.29	48	-36	45
	L	18	3.03	-51	-54	42
Posterior temporal gyrus	L	20	3.44	-57	-51	0
**Advantageous Self-maximizing Inequity condition: Inequity > Equity**						
Pallidum	R	34	3.46	24	-6	3
Ventrolateral prefrontal cortex	R	24	3.06	36	45	-3
**Advantageous Competitive Inequity condition: Inequity > Equity**						
Dorsal anterior cingulate cortex	-	49	3.39	9	39	21
Somatosensory cortex	R	17	3.12	36	-3	27

Note. p < .005 uncorrected, > 10 voxels; L/R=Left/Right; k=cluster size in 3×3×3mm voxels; Z=z-score; MNI coordinates =xyz voxel coordinates in MNI space of the peak voxel.
