# Supplementary material for: Neural Correlates of Advantageous and Disadvantageous Inequity in Sharing Decisions
Source: PLoS One. 2014 Sep 19;9(9):e107996. doi: 10.1371/journal.pone.0107996 (PMC4169616; doi:10.1371/journal.pone.0107996)
Supplement: Table S2 — Regions of neural activation from whole-brain regression analyses with frequency of inequity choices per game as a regressor. (PDF) [file pone.0107996.s002.pdf]

Table S2. Regions of neural activation from whole-brain regression analyses with frequency of inequity choices per game as a regressor.

Anatomical region	L/R	Voxels	z	MNI coordinates		
				x	y	z
**Positive correlation with frequency inequity choices**						
Advantageous Self-maximizing Inequity – null contrast						
Posterior cingulate cortex	-	17	3.71	-3	27	36
Dorsolateral prefrontal cortex	R	10	3.47	27	42	36
Advantageous Competitive Inequity – null contrast						
Cuneus	L	27	3.86	36	21	15
**Negative correlation with frequency inequity choices**						
Advantageous Competitive Inequity – null contrast						
Cuneus	R	18	3.91	18	-78	27
Putamen	R	12	3.26	30	9	0

Note. $p < .001$ uncorrected, > 10 voxels; L/R=Left/Right; k=cluster size in 3×3×3mm voxels; *Z*=z-score; MNI coordinates =xyz voxel coordinates in MNI space of the peak voxel.
