# Supplementary material for: Neural Correlates of Advantageous and Disadvantageous Inequity in Sharing Decisions
Source: PLoS One. 2014 Sep 19;9(9):e107996. doi: 10.1371/journal.pone.0107996 (PMC4169616; doi:10.1371/journal.pone.0107996)
Supplement: Text S1 — Task instructions for the allocation game. (PDF) [file pone.0107996.s003.pdf]

Task instructions for the allocation game (Güroğlu, Will, & Crone, 2014)

In this 'Allocation Game' you may decide how coins will be divided between you and another player. You can always choose between two options to divide the coins. This is what the game looks like [slide show with an example of two buckets with red and blue coins in each]. The red coins are for you and the blue coins are for the other player. In this example you see on the left side a bucket with one red coin. You receive one coin and the other player receives no coins. On the right side you see a bucket with one red and one blue coin. You receive one coin and the other player also receives one coin. Here is another example: on the left side you see a bucket with one red and one blue coin. You receive one coin and the other player receives one coin. On the right side you see a bucket with one red and two blue coins. You receive one coin and the other player receives two coins. You may choose how the coins will be distributed. Press the button number 1 using your right index finger to choose the left option and button number 2 using your right middle finger for the option on the right. Be careful: you have five seconds to make a decision. At the end of the game the computer will choose several random trials to determine how much each player has earned. These trials determine what you earn, but also how much the other players will earn. In other words, your choices have consequences on how much everyone will earn at the end of the game. Once you have made a choice, your choice will be encircled in red on the screen. In this example, the right option is chosen [slide showing right bucket with one red and two blue coins being encircled in red]. So in that case you would earn one coin and the other player would earn two coins. You will play each round with another anonymous player. These players are not present today and have agreed to participate in the experiment on a future testing day. On each trial you see your name in red above on the left; the name of the other player will appear in blue below on the left side. If you have questions, you may ask them now. If you don't

have any other questions, you will now play several practice trials with no human participant as the second player, but a computer.
